# Effect of Pre-Wetting Recycled Mortar Aggregate on the Mechanical Properties of Masonry Mortar

**DOI:** 10.3390/ma14061547

**Published:** 2021-03-22

**Authors:** René Sebastián Mora-Ortiz, Ebelia Del Angel-Meraz, Sergio Alberto Díaz, Francisco Magaña-Hernández, Emmanuel Munguía-Balvanera, Mayra Agustina Pantoja Castro, Justino Alavez-Ramírez, Leobardo Alejandro Quiroga

**Affiliations:** 1 División Académica de Ingeniería y Arquitectura (DAIA), Universidad Juárez Autónoma de Tabasco (UJAT), Carretera Cunduacán-Jalpa de Méndez km. 1, Cunduacán, Tabasco 86690, Mexico; ebelia.delangel@ujat.mx (E.D.A.-M.); alberto.diaz@ujat.mx (S.A.D.); francisco.magana@ujat.mx (F.M.-H.); emmanuel.munguia@ujat.mx (E.M.-B.); mayra.pantoja@ujat.mx (M.A.P.C.); lquiroga@ujat.mx (L.A.Q.); 2 División Académica de Ciencias Básicas (DACB), Universidad Juárez Autónoma de Tabasco (UJAT), Carretera Cunduacán-Jalpa de Méndez km. 1, Cunduacán, Tabasco 86690, Mexico; justino.alavez@ujat.mx

**Keywords:** recycled fine aggregate, recycling, sustainable construction, mortar, pre-wetting

## Abstract

In this research we evaluated the use of recycled fine mortar aggregate (RFMA) as a fine aggregate for new masonry mortar creation. The pre-wetting effect on the aggregate before creating the mixture was analyzed as a method to reduce its absorption potential. A control mixture of conventional mortar and two groups of recycled mortars were designed with a partial replacement of natural sand by RFMA (pre-wetted and not pre-wetted) performed in different proportions. The results established that the pre-wetting process allows a reduction in the amount of water required during the creation of new mixtures, regulating the water/cement (W/C) ratio and improving the properties of recycled mortars such as air content, fresh and hardened densities, and compressive and adhesive strength for all substitution levels. Mortar made with a 20% substitution and pre-wetted until it was at 67% of its absorption capacity displayed adhesive values higher than the ones shown by the reference mortar. The pre-wetting process proves to be an easy performance technique; it is inexpensive, environmentally friendly, and the most valuable fact is that specialized equipment is not necessarily needed. This process is the most profitable option for improving RFMA exploitation and reuse.

## 1. Introduction

At the present time, the proper management of solid waste from construction and demolition (C & DW) is an important subject due to its environmental and economic implications. Reusing these materials in the creation of concrete or masonry mortar mixtures increases their useful life and reduces their excessive accumulation in landfills, in addition to contributing to the protection of natural aggregate banks. There are two types of recycled aggregates (RAs): recycled thick aggregate (RTA) and recycled fine aggregate (RFA). The latter can be obtained from concrete rubble, ceramic, or mortar. The use of RFA has been limited due to its lower density values and because it has higher water absorption than sand. This has caused some researchers not to recommend its use in the preparation of concrete [1,2]. However, several studies [3,4,5] have demonstrated that it is possible to reuse RFAs during the creation of concrete mixes in percentages of substitution of sand no greater than 20% without significantly affecting its properties. 

Substitution of natural sand with RFA in mortar mixes represents a better alternative than substitution in concrete mixes because of the differences between structural requirements. The use of RFA generated through concrete debris (recycled fine concrete aggregate, RFCA) has been examined by many researchers [6,7,8,9], demonstrating that this aggregate may substitute the sand in percentages lower than 20% in dry weight. However, research supporting the reuse of RFAs generated from mortar debris (recycled fine mortar aggregate, RFMA) is scarce. This is because compared to natural aggregate (NA), RFMA has high a porosity, low density, and high-water absorption potential [10]. These characteristics have limited the recycling of this material, which has contributed to its excessive accumulation in sanitary landfills, the establishment of clandestine landfills, and overexploitation of natural banks of materials [3]. Despite it being an RA of lower quality than that obtained from concrete, in the last decade, various researchers have analyzed its use. For example, Jiménez et al. [11] used RFA constituted from ceramic and masonry mortar in substitution of NA. The results exhibit that mortar properties are not significantly affected (in its fresh and hardened state) if a proportion of 40% of this type of RFA is replacing natural sand. Silva et al. [12] made similar conclusions concerning substitution of the NA for RFA obtained from bricks or red clay tiles, highlighting that the new mortar enhances its properties, such as compressive strength, in contrast with the control mortar. 

The main problem with recycled fine mortar aggregates (RFMA) is their high-water absorption, which is an intrinsic property of this kind of material, since this type of RFA is formed by natural aggregates with adhered mortar [13,14,15,16,17]. The porosity of this bonded mortar, as well as the interfacial transition zone (ITZ) that it forms with the natural aggregate, generate a high potential for water absorption in the RFA. This parameter is decisive in the quality of recycled mortar, since it causes the RFA to absorb water from the mix, modifying the water/cement ratio (W/C) and later producing mortars with low resistance and short durability [18,19]. 

There are two methods used to reduce the water absorption potential of the RFA: increasing the water required during the mix [11,12,20] and pre-wetting the RA before mixing [21,22,23,24,25]. Some researchers [26,27] have used RA pre-wetting without altering the amount of mixing water, obtaining slight improvements in workability and the values of resistance to compression of concrete samples. Several studies [21,24,28] have demonstrated that the pre-wetting method improves the performance of mortars and recycled concrete. Some researchers [29,30,31] recommend that it is necessary to pursue better results to obtain pre-wetted the aggregates up to 80% of the total capacity absorption of RFA. 

Despite the reported benefits of pre-wetting in the preparation of masonry mortar mixtures, this method has not been widely studied with recycled aggregates from mortar elements (RFMAs). Therefore, this investigation aimed to analyze the effects of pre-wetting the RFMA as an alternative for reducing its excessive potential for water absorption, and thus be able to take advantage of this RA during the creation of new mortar mixtures. Additionally, the following were evaluated: (i) the effects of the gradual substitution of natural aggregate (NA) with RFMA; and (ii) the separate performance of two types of this recycled aggregate: one obtained through the conventional demolition process and the other through a deconstruction process. With all of the above, it is intended to encourage the recycling of this RA, while contributing to improving the environment and promoting the development of sustainable construction. Even when the pre-wetting technique has already been used in recycled mortar mixtures with RFA obtained from concrete debris, this research confirms that this technique also produces good results if applied to RFMA, a material that has conventionally been discarded. 

## 2. Materials and Methods

### 2.1. Recycled Fine Mortar Aggregates 

Recycled fine aggregates (RFA) used in this research were obtained during the demolition process of a perimeter wall built with mortar blocks. The perimeter wall was part of an office building located in the city of Villahermosa, Tabasco, Mexico, which was demolished to expand the parking lot area. To determine the benefits of using a deconstruction process in the quality of RAs and consequently on the quality of recycled mortars, two strategies were applied: (1) demolition process for the first section of the perimeter wall following the conventional process (usually established by the contractor), and (2) demolition process for the second section of the perimeter wall following a deconstruction process (also coordinated by the contractor, but in this strategy, looking for RAs without pollutants). As a result of the strategies applied, we obtained two types of recycled fine aggregates: the RFA product of a conventional demolition process, and the RFA* product of a deconstruction process. 

The deconstruction process applied in this research is reported in detail in Mora-Ortiz et al. [19] and considered the following: (i) estimating the quantity of debris to obtain, (ii) locating the cleanest place to save the debris, (iii) removing the paint from the surface to demolish (as much as is possible) using hand tools, (iv) demolishing the wall with hand tools, and (v) crushing, screening, and storing the material in a clean container. The conventional demolition process consisted of (1) demolishing the wall of prefabricated elements with hand tools, and (2) storing the debris in a clean container. 

### 2.2. Characterization of the Materials

In this research, we used river sand as the natural aggregate (NA). The characteristics of NA and RAs are shown in Table 1 along with their respective reference standards. It is observed that the sand equivalent values between the three aggregates are similar, while the recycled aggregates exhibit lower dry density than the natural aggregate (NA). It is observed that water absorption capacity is higher in RAs than in NA [14,23]. 

**Figure 1:**
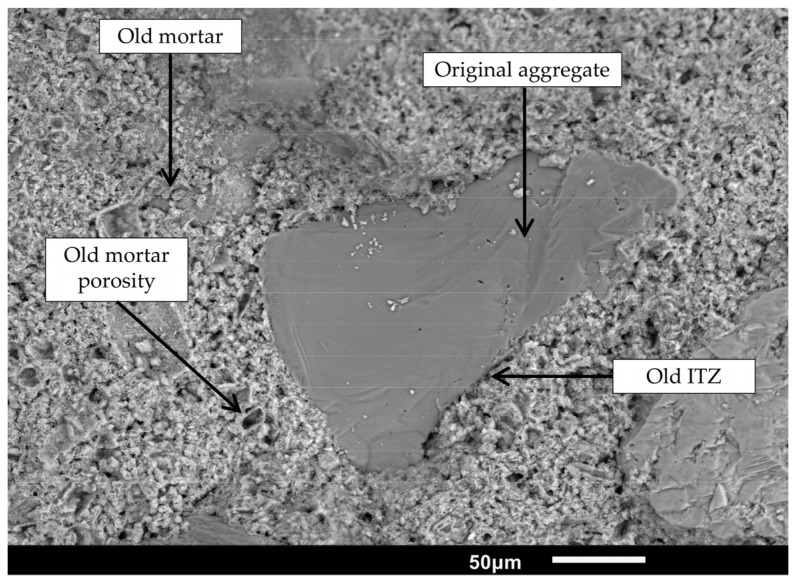
SEM photomicrograph of RFA* to 50 µm.

The high-water absorption of RAs is due to a large number of pores in the old mortar paste and the presence of the interfacial transition zone (ITZ) between the old mortar paste (old ITZ) and natural sand (original aggregate). Figure 1 shows a scanning electron microscope (SEM) photomicrograph of the RFA* using a JEOL JSM6010LA electron microscope (Boston, MA, USA). The image was taken at a 20 kV accelerating voltage in high-vacuum conditions. In this figure, the porosity of the old mortar paste and the old ITZ can be observed.

An important aspect to highlight is that the absorption potential of RFA is greater than the one shown by RFA*. The last is due to the impurities (like remnants of paint) that RFA has due to the nonexistence of a deconstruction process. 

During the characterization of the aggregates, no contaminating agent was detected (like wood and metals) that could negatively influence the properties of the mortars. It is observed that all the aggregates show optimal values regarding the content of sulfates and chloride and that among the RAs, the RFA shows slightly higher values of these parameters. This is because the RFA is the product of conventional demolition. 

The characterization of RAs used in this paper highlights the importance of establishing adequate deconstruction processes to obtain quality RAs since, as observed, the aggregates obtained with a planned demolition (RFA*) resulted in lower amounts of impurities (Table 1).

**Figure 2:**
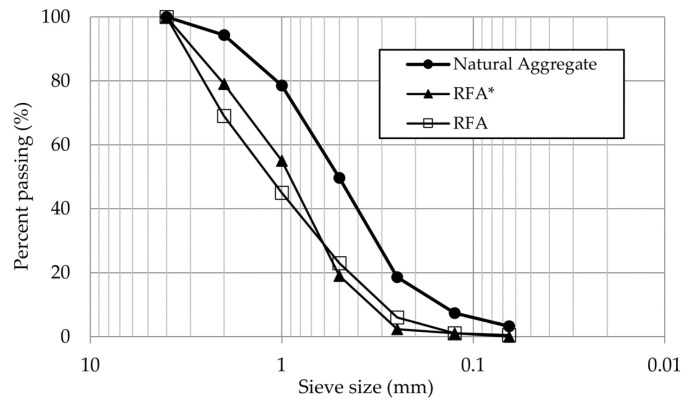
Granulometric curves of aggregates.

Figure 2 shows the granulometric curves of NA and RAs built following the procedure described in the standard UNE-EN 933-1:2012 [32]. 

**Figure 3:**
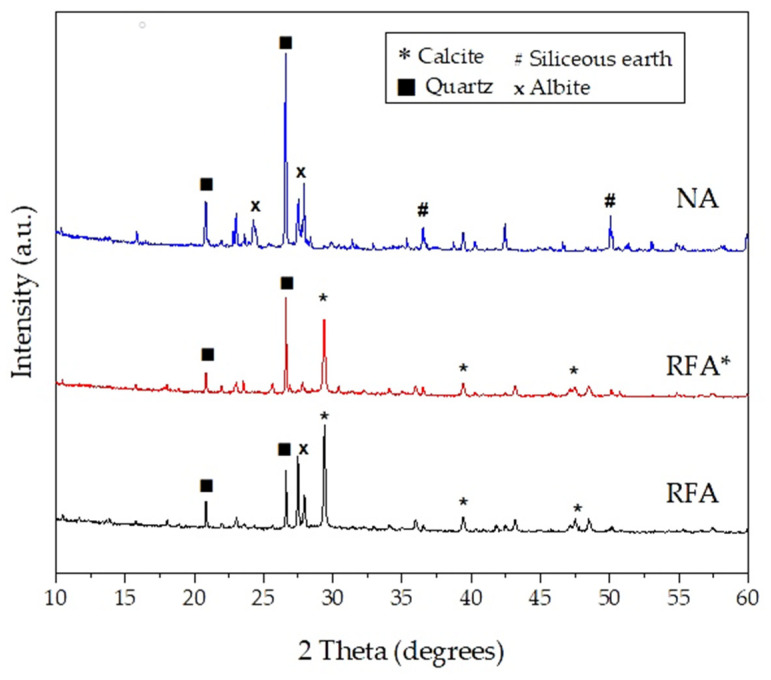
Diffraction patterns for the aggregates.

To identify the mineral phases, X-ray diffraction was applied to powders of the aggregates using Cu Kα radiation, and the diffraction patterns of the samples are shown in Figure 3. The X-ray detector was an Advance Eco model from Bruker (Coventry, England). The main crystalline phase identified for all aggregates was quartz, followed by calcite and albite, the last one being a feldspar mineral (NaAlSi_3_O_8_). All the samples have crystalline structures in nature with absences of amorphous structures. Siliceous earth (also known as Neuburg siliceous earth) is a mixture of corpuscular silica (SiO_2_) and lamellar kaolinite (Al_2_Si_2_O_5_(OH)_4_).

In Figure 3 it is observed that the aggregates exhibited similar X-ray diffraction patterns. The mineral phases exhibited by the RAs in the study depend mainly on the natural aggregate with which the mortar blocks that constituted the perimeter wall (original mortars) were manufactured. For this research, the NA used and the natural sand with which the original mortars were manufactured come from the same region. Thus, its X-ray diffraction patterns are similar. 

The cement brand used in this research was Cemex, PPC 30R type (Monterrey, Mexico), and meets international standards [36,37]. The chemical composition of the cement described before is shown in Table 2.

### 2.3. RFA Pre-Wetting Method

The pre-wetting method used was based on the procedures described by Fonseca et al. [38] and Cuenca-Moyano et al. [24], which are as follows: in a standard mixer the RAs and distilled water were mixed at low speed for five minutes; subsequently, the RAs were left to rest submerged for ten minutes; at the end of this time they were removed from the water and allowed to drain before use.

To determine the influence of pre-wetting of RAs on the mechanical properties of masonry mortars, three pre-wetting methods were used, all of them described in the literature consulted (Table 3). 

In the Pw-92 method, the RA was pre-wetted to 92% of its absorption capacity, WA_24h_ (100% of the absorption capacity of an aggregate is reached by submerging it in water for 24 h). For the Pw-80 and Pw-67 methods, RAs were pre-wetted to 80% and 67% of their absorption potential (WA_24h_), respectively. Many researchers suggest avoiding the 100% pre-wetting of the RA’s absorption capacity, because it may cause excessive mortar bleeding, among other problems [27,40].

### 2.4. Mixes

To achieve the objectives of this research, first, a mixture of conventional mortar (sand + cement + water) with NA was elaborated with a cement–sand proportion of 1:4 (dosage by weight). The project consistency was 175 ± 5 mm. This control mixture was used as a reference for the rest of the mixtures; that is, all the mixtures made in this investigation were manufactured following the same proportions. Additionally, two families of mortar were proposed: in one, the NA was partially substituted by RFA, while in the other, NA was replaced by RFA*. For these families, four proportions of replacement of NA by RA were established as a percentage of dry weight: 20%, 40%, 60%, and 100% [11,22,24,41]. 

Table 4 shows the manufacturing and proportioning data for the mixtures used. In the name of the type of mortar, the first letter refers to the type of recycled aggregate with which that mortar was manufactured: R for mortar made with RFA, and *R for those manufactured with RFA*. The number next to the first letter refers to the percentage of pre-wetting: 0 (without pre-wetting), 67, 80, and 92%. Finally, the number after the hyphen indicates the percentage of substitution of NA by the RA: 20, 40, 60, and 100%. For example, the R67-40 mortar was made with the recycled aggregate RFA, which was pre-wetted to 67% of its absorption potential, and the NA was replaced by RFA in a 40% substitution. For its part, the *R0-60 mortar was manufactured with the recycled aggregate RFA*, it did not receive pre-wetting and the NA was replaced by 60%. The aggregates that did not receive pre-wetting were used with their natural moisture (2.3 ± 0.2). To promote the project consistency over all the mixtures, it was necessary to adjust the amount of water during the mix (Table 4). A total of 33 different types of mortar were analyzed. 

All the mixtures were made following strictly the same procedure: (i) the cement and the fine aggregate were mixed dry (including those aggregates that were pre-wetted) at low speed until a homogeneous mixture was achieved, (ii) the water was then added for 20 s while the mixer was still mixing the solid elements, (iii) the three components continued to mix at low speed for three minutes.

### 2.5. Rehearsal Program

Table 5 shows the properties evaluated and the corresponding standards applied. 

## 3. Results and Discussion

### 3.1. Fresh Mortar

#### 3.1.1. Bulk Density

**Figure 4:**
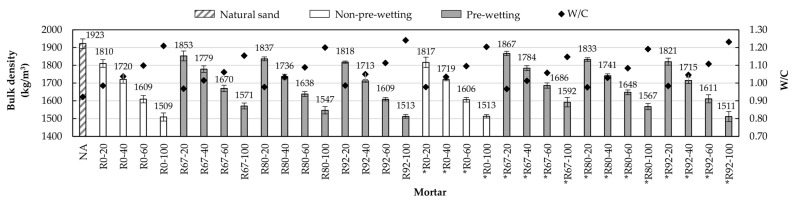
The substitution effect of natural aggregate (NA) by RA in fresh state density.

Figure 4 shows bulk density values for the mortars analyzed along with their water/cement ratio. It is possible to observe that in general, for all mortar series, according to the amount of RA that increases in the mixtures, the bulk density decreases, so that for each series, the mortar with the highest density was a 20% substitution, and the mortars that exhibited the lowest density were those in which 100% of the NA was substituted by RA. The latter is due to the high absorption potential and the low density of RA [3,19,48]. The first characteristic causes it to reach the project consistency (175 ± 5 mm) and more water is required in the mix, which in turn causes an increase in the water/cement ratio, making the mortar less dense. Other researchers [12,49] have observed the same behavior in mortars elaborated with RFA obtained from concrete and ceramics.

Both families of mortar with recycled aggregates (RFA and RFA*) exhibited very similar behavior, however, the mortars made with RFA* showed slightly higher density values than their homologous mortars made with RFA. It is observed that the differences in densities between homologous mixtures do not exceed 2%. For example, mortar *R67-20 (mortar with the highest apparent density of all) is 0.75% denser than its homologous mortar R67-20 (the second mortar with the highest apparent density). This behavior is because RFA* has a lower water absorption potential than RFA, which translates into mortars with a lower water/cement ratio.

The mortars with the lowest density values were those in which the recycled aggregates did not receive pre-wetting (R0 and *R0). This is because by not pre-wetting the RAs, the amount of water required to reach the consistency of the project is greater, so that the water/cement ratio increases. In both families of mortar, the pre-wetting method generated the lowest water/cement ratios and therefore the mortars with the highest densities were Pw-67 (67% of WA_24_). It is observed that if the amount of water used in pre-wetting increases, a reduction in the density of the mortars is generated so that mortars that were made with RAs pre-moistened to 92% (Pw-92) of their capacity of absorption showed the lowest densities of the pre-wetting methods.

**Figure 5:**
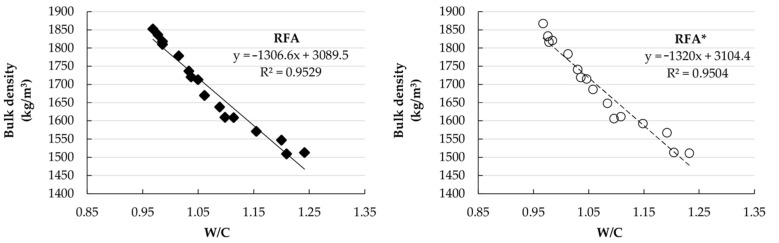
Correlation between the density of fresh mortar and the modifications in the water/cement (W/C) ratio for RFA and RFA*.

Figure 5 shows the correlation between values from the bulk density of fresh mortars and the modifications in the water/cement ratio. It is possible to observe a good correlation index for both types of recycled fine aggregate (RFA and RFA*).

#### 3.1.2. Air Content

This property has a vast influence on the development of the compressive strength and durability of masonry mortars. Despite its importance, currently, building regulations do not specifically limit values for this important parameter. In this investigation, the optimal air content in masonry mortar mixes was in the range of 5 to 20%, this same range is suggested by other researchers [22,24]. 

**Figure 6:**
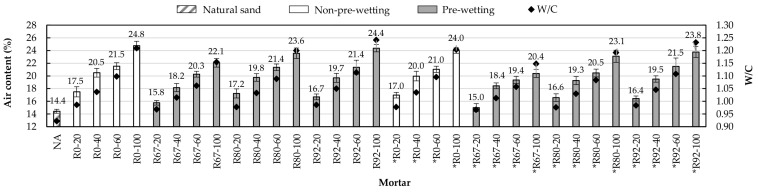
Values of air content and W/C ratio.

Figure 6 shows the air content value of the analyzed mortar mixtures as well as their corresponding value of water/cement ratio. It is remarkable to show that all the mixtures with pre-wetting and with percentages of substitution of NA by RA less than 60% complied with the reference range adopted in this investigation. However, in the mortar mixtures that did not receive pre-wetting, only the substitution percentage of 20% met the reference range. 

Mortars made with partial substitution of NA for RA exhibited a higher air content than the control mortar. It is noted that in all mortar series made with both RAs, the air content increased accordingly to the water/cement ratio increase. Thus, the mortars with the highest and lowest air content were those manufactured with NA substitutions of 20 and 100%, respectively. 

The mortar families made with RFA and RFA* (R and *R, respectively) presented values of air content like each other, however, the mortars made with RFA* showed values of air content slightly smaller than their RFA-made homologous mortars. For example, the air content of mortar *R67-40 was 1.1% less than mortar R67-40. This is due to the lower absorption potential of RFA*.

The results show that mortars made without pre-wetting the RA (R0 and *R0) are those with the highest air content values. The pre-wetting method that produced mortars with the lowest air content was Pw-67%. It is observed that if the pre-wetting percentage increases, the water/cement ratio rises, causing an increase in air content.

**Figure 7:**
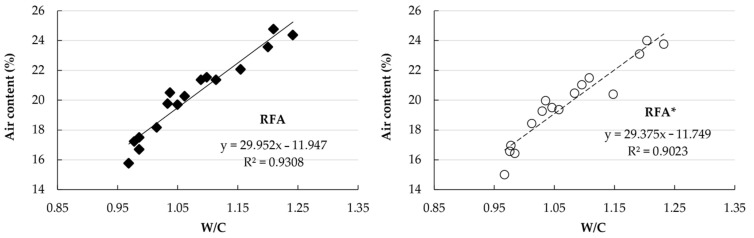
Correlation of air content and W/C ratio.

Figure 7 shows the correlation between air content and the analyzed mortars’ water/cement ratio.

The results demonstrate the influence that water/cement ratio exerts on the properties of masonry mortar in a fresh state: if the water/cement ratio increases, the air content increases too, but the bulk density decreases. It can be observed that among the pre-wetted mortars those with a similar water/cement value have similar densities in a fresh state and similar air content. These results are consistent with what has been observed by other researchers [50].

### 3.2. Hardened Mortar

#### 3.2.1. Dry Bulk Density

**Figure 8:**
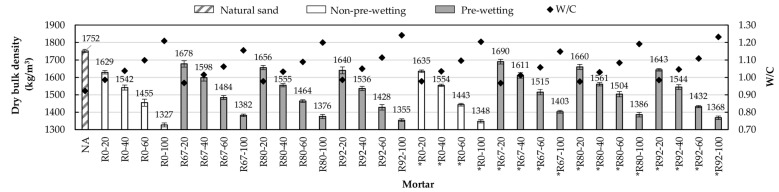
Dry bulk density and water/cement ratio.

The dry bulk density values of the mortars under study, along together with their respective water/cement ratios, are shown in Figure 8. 

It is observed that the dry bulk density decreases with the percentage of substitution of NA by RA, and the water/cement ratio increases. This behavior is repeated for all the series with substitution of the NA for either of the two RAs and is consistent with the observations made by other researchers [11,51,52].

In general, mortars made with RFA* exhibited slightly higher dry bulk density values than their counterparts made with RFA. This difference did not exceed 3%. 

The pre-wetting method that produced the densest mortars was Pw-67 (67% of WA_24_). According to the pre-wetting percentage increase, the dry bulk density of the mortars decreased. Therefore, of the pre-wetting methods used, Pw-92 (92% of WA_24_) was the method that produced the lowest density mortars. Once again, of all the samples analyzed, the mortars with the lowest density were those in which the RAs did not receive pre-wetting. 

**Figure 9:**
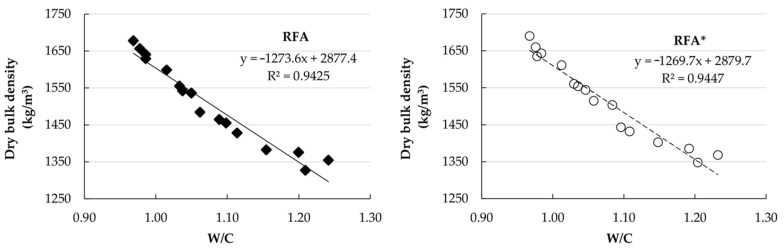
Correlation between dry bulk density and water/cement ratio.

**Figure 10:**
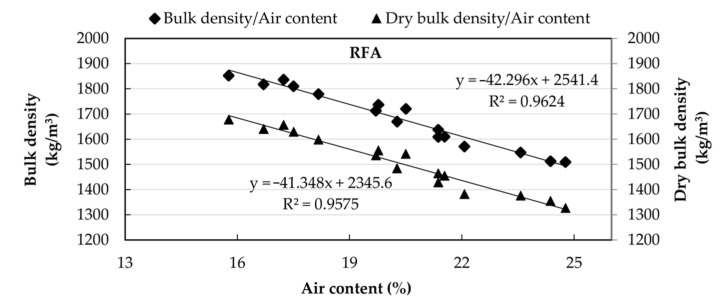
Correlation between densities (fresh and dry state) and the air content in mortar made with RFA.

**Figure 11:**
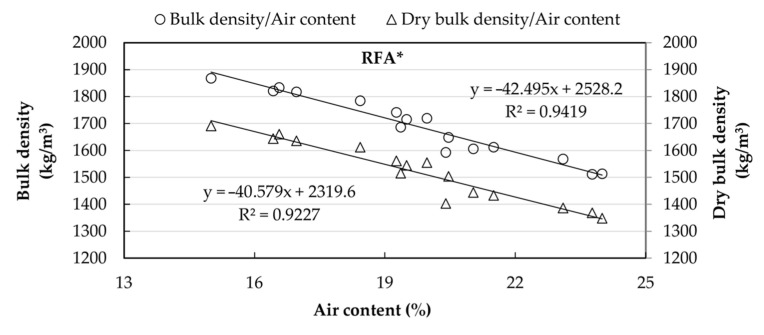
Correlation between densities (fresh and dry state) and the air content for in mortar made with RFA*.

Figure 9 shows the correlation between dry bulk density and water/cement ratio for both RAs. Figure 10 and Figure 11 show an inverse linear relationship between the bulk density of fresh mortars, the air content, and the dry bulk density in the mortars elaborated with RFA and RFA*, respectively, such that, as the air content increases, the densities decrease. 

#### 3.2.2. Compressive Strength

**Figure 12:**
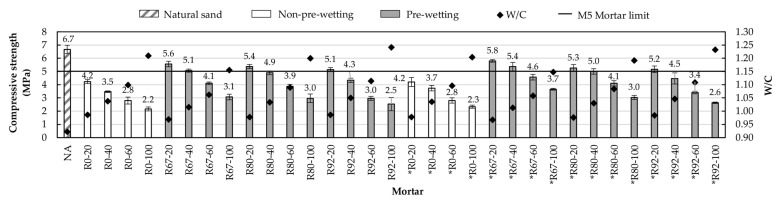
Values of compressive strength and W/C ratio.

This is a very interesting property because the durability of masonry mortars is linked to it. The determination of this property was conducted by the method established in the standard UNE-EN 1015-11 [45]. Figure 12 shows the values of compressive strength and the corresponding water/cement ratios of the mortars studied. It is possible to notice that all mortar series show a trend like the one observed in the properties for the fresh state: a decrease in its value as a result of the increase in the water/cement ratio caused by the increase in the percentage of substitution of NA by RA, the above agrees with the results reported in other investigations [21,31,53]. 

Comparing the families of mortar with the replacement of natural aggregate by RA, it is observed that the mortars made with RFA* showed greater resistance to compression than their homologous mortars made with RFA. This is because the former have the lowest water/cement ratio. 

It is observed that the reference mortar exceeded the resistance required for a class M5 mortar, but no mortar without pre-wetting of the RA reached this requirement. The results show that pre-wetting RAs generated an increase in the compressive strength values of the mortars. For example, for a 20% RA content, it is observed that pre-wetting the aggregate to 67% of its absorption capacity produces an increase in strength of around 25% due to mortar with aggregates without pre-wetting. 

Of the pre-wetting methods used, Pw-67 demonstrated the best results. Again, if the percentage of pre-wetting increases, the water/cement ratio increases too, but by contrast, the compressive strength values of the mortars decrease. Of the mortars with pre-wetting of 67% of their absorption potential, only the replacement proportions of 20 and 40% met the minimum resistance established for mortars of class M5. It is important to note that the latter was achieved in both families of mortars, which indicates that in terms of resistance to compression there are no considerable differences between using any of these additions. It is observed that between homologous mortars that satisfy the minimum resistance of 5 MPa, the difference between the strengths does not exceed 7%. Regarding the rest of the pre-wetting methods used, it is observed that in both families of mortar (RFA* and RFA), only those with 20% substitution exceeded the established minimum resistance.

#### 3.2.3. Adhesive Strength 

**Figure 13:**
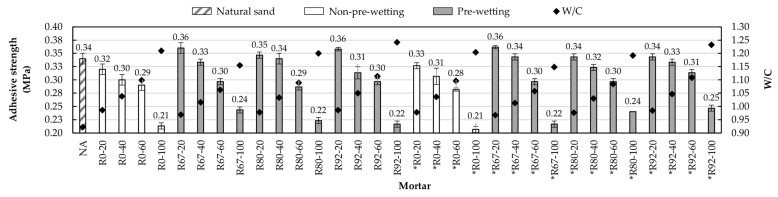
Adhesive strength and water/cement ratio.

Figure 13 shows the adhesive strength values of the mortars studied, as well as their corresponding water/cement ratios. The determination of this property was conducted using the method established in the standard UNE-EN1015-12 [46]. The results made it clear that the increase in the substitution of NA for RA affects this important parameter of masonry mortars. However, an interesting point is that all mortars with 20% NA substitution exhibited adhesive strength values that were very close to or greater than the reference mortar. The above indicates that for the mentioned substitution percentage there is no negative effect from adding RAs to the mortar mixes [6,7,11,53]. This increase in adhesive strength is due to the penetration of the cement paste into the pores of the RA, acting as a microstructural anchor, and with the above, a high-quality interfacial transition zone is formed [20].

From analyzing the results it is observed that the mortars manufactured with pre-wetting exhibited higher adhesion strength values than the mortars that were not pre-wetted. However, pre-wetting methods did not show the trend observed in the properties analyzed above; that is, the adhesive strength did not decrease as clearly with increasing the amount of pre-wetting water. The three methods of pre-wetting generated very similar adhesion values in both mortar families (RFA and RFA*). 

Regarding the type of aggregate with which the mortars were manufactured (RFA and RFA*), no clear difference was observed between using one or the other; both families showed similar values between their homologous mortars. For example, R67-40 mortar has an adhesion value of 0.33 MPa, and *R67-40 mortar exhibits 0.34 MPa of adhesive strength (2.9% difference).

#### 3.2.4. Water Absorption Due to Capillary Action of Hardened Mortar

This property is one of the main indicators of the durability of the mortar because high absorptions are related to low durability [6,24]. This important parameter depends on the pore network developed in the mortars; that is, the more developed the pore network, the greater its absorption. Mortars with high absorption allow the passage of water and this, in turn, can transport substances that severely affect the durability of the mortar. Therefore, the water absorption values should be as low as possible.

**Figure 14:**
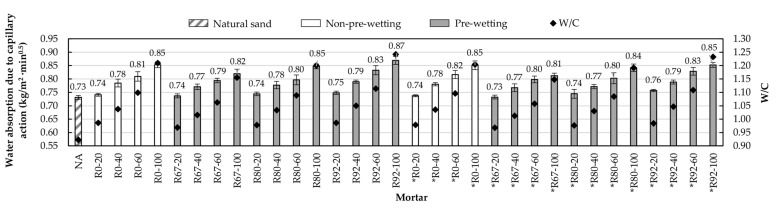
Water absorption due capillary action values and water/cement ratio.

The determination of this property was conducted using the method established in the standard UNE-EN1015-18 [47]. Figure 14 shows the values of the water absorption of the mortars analyzed together with their water/cement ratio. In all the series analyzed, it is observed that when the RA proportion increases and consequently the water/cement ratio increases, the absorption value increases [11,22]. It is important to highlight that the water absorption value exhibited by mortars was not affected by the type of pre-wetting used or by the type of aggregate with which the mortar was manufactured. It can be observed that mortars with similar water/cement ratios have similar water absorptions. 

**Figure 15:**
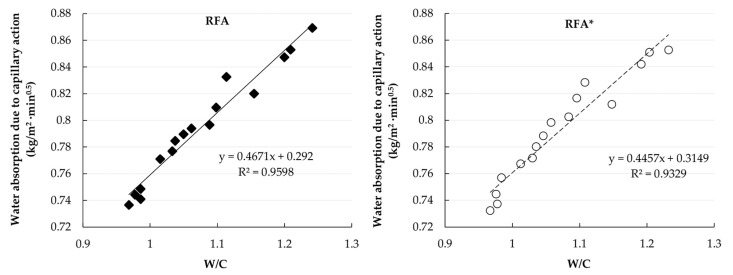
Correlation between water absorption and water/cement ratio.

The relationship between water absorption due to capillary action and water/cement ratio is shown in Figure 15.

## 4. Conclusions

Concerning the gradual replacement of NA by RA, a very well-defined trend was observed in all recycled mortars: increasing the proportion of RA in mortar mixes is detrimental to mortar properties. This is due to the low density of RA regarding NA, but is mainly due to its high absorption value. Experimental results allow us to conclude that the properties of recycled mortars are a function of the proportion of substitution of NA by RA as well as the water/cement ratio. 

Although mortars made with RFA* had slightly better results than mortars made with RFA, the truth is that both families of mortar exhibited very similar physical–mechanical behavior. In all the properties analyzed, for both fresh and hardened mortars, the values between homologous mortars from both families were always very close. However, although the deconstruction method used to obtain the RFA* is simple, it generated an 11% increase in demolition costs. Therefore, at least for the aggregates used in this research, the cost–benefit relation indicates the RFA aggregate as the best option. It is important to note that neither of the aggregates were obtained from a construction waste deposit but were obtained directly from the demolition site. The latter was a decisive aspect in the quality of both types of aggregate and in the results they showed. 

Comparing the recycled mortars with pre-wetting of the RAs with those mortars in which the RAs were used with their natural moisture, it was found that the pre-wetting generated substantial improvements in the mortar properties. This is because pre-wetting the aggregates prior to mixing reduces the transfer of water between the cement paste and the RA, so no more water is required to achieve project consistency and the water/cement ratio does not increase. The results show that the optimal percentage of pre-wetting water was 67% of the total absorption potential of the RAs (Pw-67). This pre-wetting method allows the replacement of NA by RFMA in a proportion up to 20% without a significant effect on the mortar properties. 

The use of pre-wetting will contribute to the reuse of this type of RA in conventional masonry mortar applications such as block bonding and rendering (indoor and outdoor), as well as in the preparation of precast elements. For its implementation on-site, it would be necessary to allocate 15 min for the pre-wetting of the RA (five minutes of wetting and ten minutes of rest) before mixing with the other elements of the mortar. All of the above will contribute to reducing the accumulation of these RAs in landfills and protecting natural material banks.

## Figures and Tables

**Table 1:** Main characteristics of natural aggregate and recycled fine aggregates (RFAs).

Property	Standard	Limit Value	NA	RFA	RFA*
Fine content (%)	EN 933-1 [32]	≤30	9.12	6.53	6.50
Sand equivalent (%)	EN 933-8 [33]	No limit	95	84	83
Dry sample density (gr/cm^3^)	EN 1097-6 [34]	No limit	2.65	2.19	2.21
Water absorption (%)	EN 1097-6 [34]	No limit	1.28	7.82	7.59
Acid soluble sulphates (% SO_3_)	EN 1744-1 [35]	≤0.8	<0.010	0.0038	0.0029
Water-soluble chlorides (% Cl)	EN 1744-1 [35]	≤0.06	<0.010	0.055	0.031
Total sulphurs (% SO_3_)	EN 1744-1 [35]	≤1	<0.010	0.0041	0.0028

**Table 2:** Chemical composition of cement used (given by the fabricant).

Composition	CaO	SiO_2_	Al_2_O_3_	Fe_2_O_3_	MgO	K_2_O	Na_2_O	SO_3_
%	63	22	6	2.5	2.6	0.6	0.3	2.0

**Table 3:** Pre-wetting methods.

Pre-Wetting Method	Pre-Wetting Water			Reference
	% of WA ^a^	Value ^b^ RFA (%)	Value ^b^ RFA* (%)	
Pw-92	92	7.19	6.98	[24]
Pw-80	80	6.26	6.07	[23,29]
Pw-67	67	5.24	5.09	[24,39]

^a^ Water absorption capacity of recycled aggregate (RA), ^b^ by dry mass (g) of RA added.

**Table 4:** Mortar mixture proportions.

Mortar Type	RA Type	NA/RA (%)	NA (g)	RA (g)	CEM (g)	Pre-Wetting		Mixing Water (g)	Total Water (g)	Consistency Index (mm)	W/C
						Method	Water (g)				
Control	-	100/0	2505	0	564	-	0	520	520	175	0.922
R0-20	RFA	80/20	2004	501	564		0	556	556	170	0.986
R0-40		60/40	1503	1002	564		0	585	585	172	1.037
R0-60		40/60	1002	1503	564		0	619	619	177	1.098
R0-100		0/100	0	2505	564		0	682	682	179	1.209
R67-20	RFA	80/20	2004	501	564	Pw-67	26	520	546	172	0.969
R67-40		60/40	1503	1002	564		52	520	572	178	1.015
R67-60		40/60	1002	1503	564		79	520	599	173	1.062
R67-100		0/100	0	2505	564		131	520	651	171	1.155
R80-20	RFA	80/20	2004	501	564	Pw-80	31	520	551	174	0.978
R80-40		60/40	1503	1002	564		63	520	583	179	1.033
R80-60		40/60	1002	1503	564		94	520	614	171	1.089
R80-100		0/100	0	2505	564		157	520	677	176	1.200
R92-20	RFA	80/20	2004	501	564	Pw-92	36	520	556	174	0.986
R92-40		60/40	1503	1002	564		72	520	592	178	1.050
R92-60		40/60	1002	1503	564		108	520	628	175	1.114
R92-100		0/100	0	2505	564		180	520	700	170	1.242
*R0-20	RFA*	80/20	2004	501	564	0	30	551	551	171	0.978
*R0-40		60/40	1503	1002	564		61	584	584	174	1.035
*R0-60		40/60	1002	1503	564		91	618	618	173	1.096
*R0-100		0/100	0	2505	564		152	679	679	178	1.204
*R67-20	RFA*	80/20	2004	501	564	Pw-67	25	520	545	177	0.967
*R67-40		60/40	1503	1002	564		51	520	571	174	1.012
*R67-60		40/60	1002	1503	564		76	520	596	180	1.058
*R67-100		0/100	0	2505	564		127	520	647	177	1.148
*R80-20	RFA*	80/20	2004	501	564	Pw-80	30	520	550	170	0.976
*R80-40		60/40	1503	1002	564		61	520	581	178	1.030
*R80-60		40/60	1002	1503	564		91	520	611	172	1.084
*R80-100		0/100	0	2505	564		152	520	672	174	1.192
*R92-20	RFA*	80/20	2004	501	564	Pw-92	35	520	555	175	0.984
*R92-40		60/40	1503	1002	564		70	520	590	177	1.046
*R92-60		40/60	1002	1503	564		105	520	625	172	1.108
*R92-100		0/100	0	2505	564		175	520	695	179	1.232

**Table 5:** Properties analyzed in mortars and standards of reference.

Test	Standard	Curing Time (Days)
*Properties of fresh mortar*		
Bulk density of the fresh mortar	UNE-EN 1015-6 [42]	-
Entrained air	UNE-EN 1015-7 [43]	-
*Properties of hardened mortar*		
Dry bulk density	UNE-EN 1015-10 [44]	28
Compressive strength	UNE-EN 1015-11 [45]	28
Adhesive strength	UNE-EN 1015-12 [46]	28
Water absorption coefficient due to capillary action	UNE-EN 1015-18 [47]	28

## Data Availability

Data is contained within the article.
